# Quantitative Evaluation of Drug Resistance Profile of Cells Expressing Wild-Type or Genetic Polymorphic Variants of the Human ABC Transporter ABCC4

**DOI:** 10.3390/ijms18071435

**Published:** 2017-07-04

**Authors:** Megumi Tsukamoto, Shiori Sato, Kazuhiro Satake, Mizuki Miyake, Hiroshi Nakagawa

**Affiliations:** 1 Department of Applied Biological Chemistry, Graduate School of Bioscience and Biotechnology, Chubu University, 1200 Matsumoto-cho, Kasugai 487-8501, Japan; gr16801@isc.chubu.ac.jp (M.T.); ksatake@isc.chubu.ac.jp (K.S.); gr15020@isc.chubu.ac.jp (M.M.); 2 Department of Applied Biological Chemistry, College of Bioscience and Biotechnology, Chubu University, 1200 Matsumoto-cho, Kasugai, Aichi 487-8501, Japan; fr14042-0218@sti.chubu.ac.jp

**Keywords:** ATP-binding cassette (ABC) transporter, ATP-binding cassette subfamily C member 4 (ABCC4), drug resistance, single-nucleotide polymorphism (SNP), multi drug resistance protein 4 (MRP4)

## Abstract

Broad-spectrum resistance in cancer cells is often caused by the overexpression of ABC transporters; which varies across individuals because of genetic single-nucleotide polymorphisms (SNPs). In the present study; we focused on human ABCC4 and established cells expressing the wild-type (WT) or SNP variants of human ABCC4 using the Flp-In™ system (Invitrogen, Life Technologies Corp, Carlsbad, CA, USA) based on Flp recombinase-mediated transfection to quantitatively evaluate the effects of nonsynonymous SNPs on the drug resistance profiles of cells. The mRNA levels of the cells expressing each *ABCC4* variant were comparable. 3-(4,5-Dimethyl-2-thiazol-2-yl)-2,5-diphenyl-2H-tetrazolium bromide (MTT) assay clearly indicated that the EC_50_ values of azathioprine against cells expressing ABCC4 (WT) were 1.4–1.7-fold higher than those against cells expressing SNP variants of ABCC4 (M184K; N297S; K304N or E757K). EC_50_ values of 6-mercaptopurine or 7-Ethyl-10-hydroxy-camptothecin (SN-38) against cells expressing ABCC4 (WT) were also 1.4–2.0- or 1.9-fold higher than those against cells expressing the SNP variants of ABCC4 (K304N or E757K) or (K304N; P403L or E757K); respectively. These results indicate that the effects of nonsynonymous SNPs on the drug resistance profiles of cells expressing *ABCC4* can be quantitatively evaluated using the Flp-In™ system.

## 1. Introduction

Cancer is one of the chief causes of mortality in many developed countries. The broad-spectrum drug resistance of cancer cells varies across individuals and poses a major challenge to cancer research and treatment [1,2]. Drug resistance of cancer cells and differences in their individual levels are usually caused by the overexpression of ABC transporters and single-nucleotide polymorphisms (SNPs) in their genes, respectively. Thus, SNPs in ABC transporter genes determine the response rate to cancer chemotherapy, and development of easy-to-use and quantitative approaches for the identification of these individual-specific SNPs would help combat cancer cell drug resistance through personalized chemotherapy.

The human body expresses 48 ABC transporters, which are further divided into seven sub-families (ABCA–ABCG) based on sequence homology and protein organization [3]. The transporters play critical roles in physiological transport and export of drugs and toxic substances, wherein many endogenous and exogenous substrates are transported across membranes in an ATP-dependent manner [4,5,6,7,8,9,10]. ABCC4, located on chromosome 13q32.1 encodes for the 1325-amino acid-long human ABCC4 (MRP4), and is widely expressed in various tissues, including the liver, kidney, ovary and blood cells [11,12,13]. Since the first report in 1999 about the direct link between *ABCC4* overexpression and impaired efficacy of nucleoside-based antiviral drugs in a human T-lymphoid cell line [14], ABCC4 has been reported to transport a broad spectrum of xenobiotics, including antiviral, antibiotic, antihypertensive and anticancer drugs such as azathioprine, 6-mercaptopurine, and SN-38 [12,13,14,15,16,17,18,19,20,21,22,23,24,25]. The affinity of ABCC4 for its substrate drugs is altered by some of the ≥140 non-synonymous SNPs in *ABCC4* [13,24,25]. The SNP variants of *ABCC4* (rs11568658, 559 G > T; rs753414892, 1167 A > G; rs11568668, 1460 A > G; rs3765534, 2269 G > A; rs146708960, 2326 G > A; and rs11568644, 3425 C > T) have been suggested to be associated with reduced function of ABCC4, wherein the cellular disposition of substrates for ABCC4 was altered [13,24,25,26].

Various quantitative functional analyses of ABCC4 [wild-type (WT) or single-nucleotide polymorphisms (SNPs)] have been performed [13,24,25]. However, thus far, the drug sensitivities of cells expressing WT or SNP variants of ABCC4 have never been quantitatively evaluated, since it is difficult to control the integration number and integration site of the cDNA in the genome using traditional transfection methods for establishing cell lines expressing the exogenous gene. Unlike the traditional system, the Flp-In™ system, which is based on the Flp recombinase-mediated transfection can integrate a single copy of the cDNA into the FRT site generated in the telomeric region of the short arm of one copy of chromosome 12 in Flp-In-293 cells [27]. We have reported that the Flp-In™ system can be used to generate cell lines for quantitatively evaluating the effects of the nonsynonymous SNPs on drug resistance profiles [27,28,29,30]. Therefore, in this study, we performed a quantitative evaluation of the drug resistance profiles of the cells expressing the WT or SNP variants (M184K, N297S, K304N, P403L or E757K) of human ABCC4 using the Flp-In™ system.

## 2. Results

### 2.1. Levels of ABCC4 mRNA and Protein in Cells Established Using the Flp-In™ System

**Figure 1:**
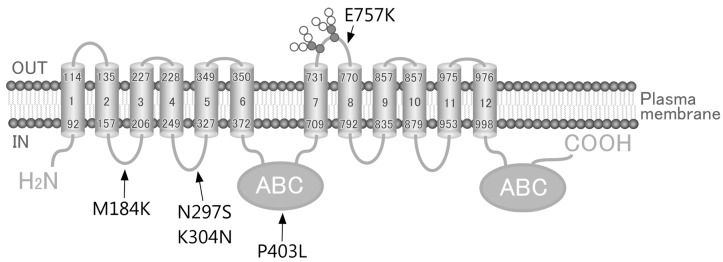
Schematic illustration of human ABCC4 and the location of its single-nucleotide polymorphisms (SNPs). Arrows, location of SNPs; ABC, ATP binding cassette (nucleotide binding domain).

In the present study, we employed Flp-In-293 cells with the Flp-In™ system to establish cells expressing WT or non-synonymous SNP variants of human ABCC4 (Figure 1 and Table 1). Flp-In-293 cells were transfected with the *ABCC4* cDNA, which integrated into the FRT-tagged genomic DNA, and were then selected using hygromycin B. The resulting hygromycin B-resistant cells were analyzed using qPCR, where the mRNA levels of *ABCC4* and *glyceraldehyde-3-phosphate dehydrogenase* (*GAPDH*) were measured. In the present study, the mRNA levels of *ABCC4* were corrected according to those of *GAPDH*, and the resulting *ABCC4* mRNA levels were compared among the established cells to evaluate the success of the Flp-In™ system.

**Figure 2:**
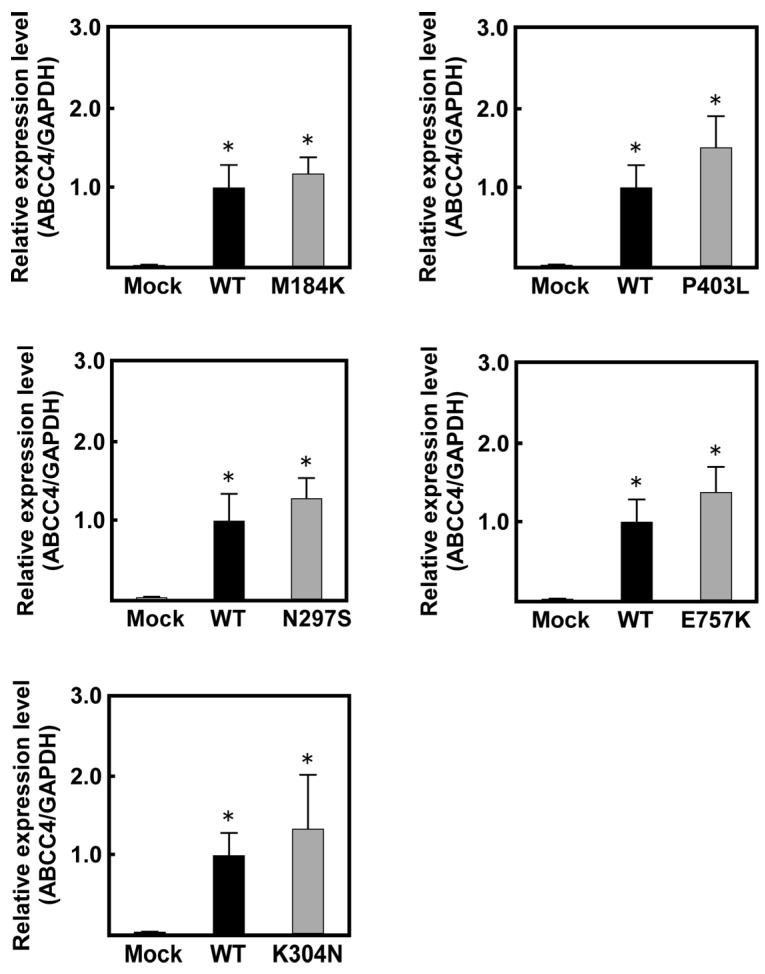
Levels of *ABCC4* mRNA in cells established using the Flp-In™ system. The levels of *ABCC4* and *GAPDH* mRNA were measured using qPCR with specific primer sets for *ABCC4* and *GAPDH*, as described in Materials and Methods. Data are calculated as ratios by referring to the *GAPDH* mRNA levels in the cells and normalized to the ratio of *ABCC4*/*GAPDH*. Data are expressed as mean values ± S.D. (*n* = 5). Statistical analyses for significance were performed using one-way ANOVA and Tukey HSD test (* *p* < 0.01 compared to the Mock group).

As shown in Figure 2, *ABCC4* mRNA levels in the cells transfected with *ABCC4* cDNA were >42-fold higher than those in Flp-In-293/Mock cells. In contrast, the levels of *ABCC4* mRNA were comparable among the cells transfected with *ABCC4* cDNA, indicating that the Flp-In™ system functioned in the cells established in the present study.

**Figure 3:**
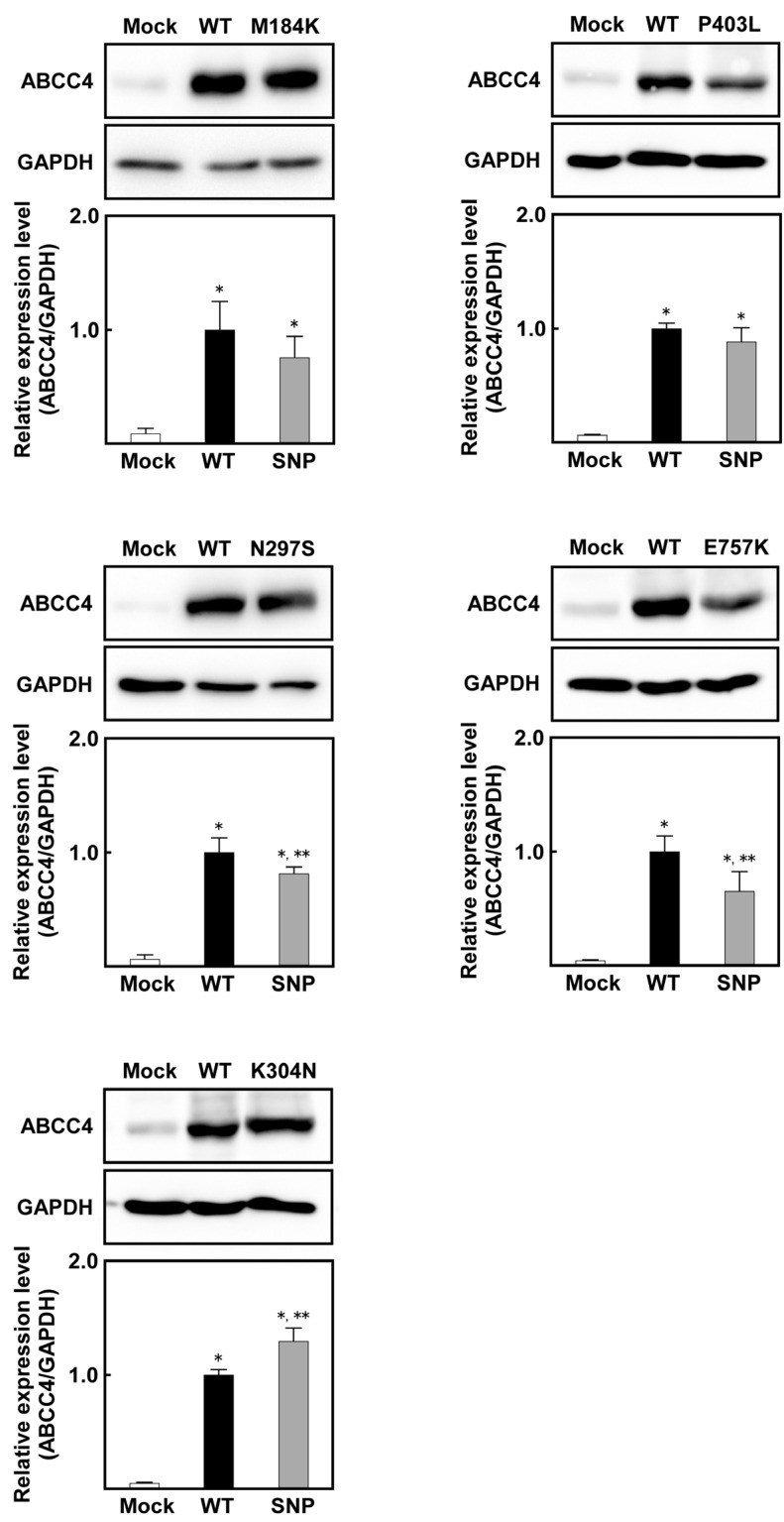
Levels of ABCC4 in cells established using the Flp-In™ system. ABCC4 and GAPDH levels were detected using western blot analysis with specific antibodies for ABCC4 and GAPDH, and their levels were measured using ImageJ (Wayne Rasband, Bethesda, MD, USA) as described in Materials and Methods. ABCC4-specific monoclonal antibody (M4I-10) or GAPDH-specific antibody was used for protein detection in PNGase F-treated cell lysate. The experiments were performed independently more than two times. Data are expressed as mean values ± S.D. (*n* = 3 or 4). Statistical analyses for significance were performed using one-way ANOVA and Tukey HSD test (* *p* < 0.05 compared to the Mock group; ** *p* < 0.05 compared to the WT).

Since qPCR clearly showed that the Flp-In™ system functioned in these cells, western blot analysis was performed to evaluate the expression of ABCC4 and GAPDH in these cells, wherein all samples were treated with PNGase F to remove the glycomoieties on ABCC4. As shown in Figure 3, the level of ABCC4 was found to correspond to that of the *ABCC4* mRNA, and the level of ABCC4 in the *ABCC4* cDNA-transfected cells was much higher than that in Flp-In-293/Mock cells. On the contrary, the levels of ABCC4 in cells expressing ABCC4 (M184K or P403L), ABCC4 (N297S or E757K) and ABCC4 (K304N) were comparable, lower and higher compared to that in cells expressing ABCC4 (WT), respectively.

### 2.2. Anticancer Drug Resistance Properties of Cells Established Using the Flp-In™ System

**Figure 4:**
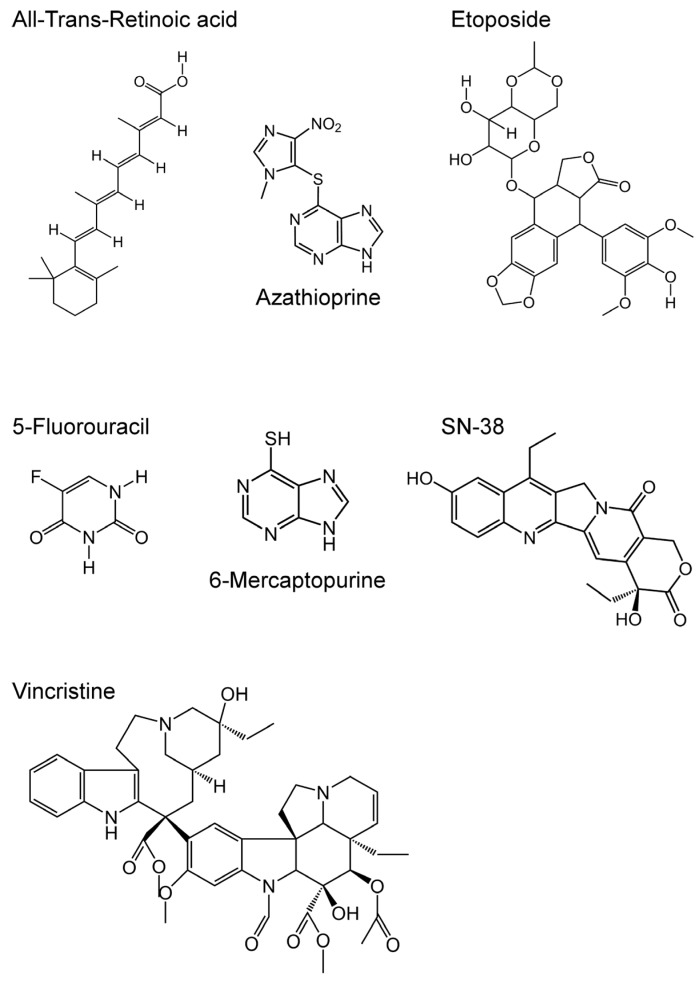
Structures of the anticancer drugs tested in the present study.

**Figure 5:**
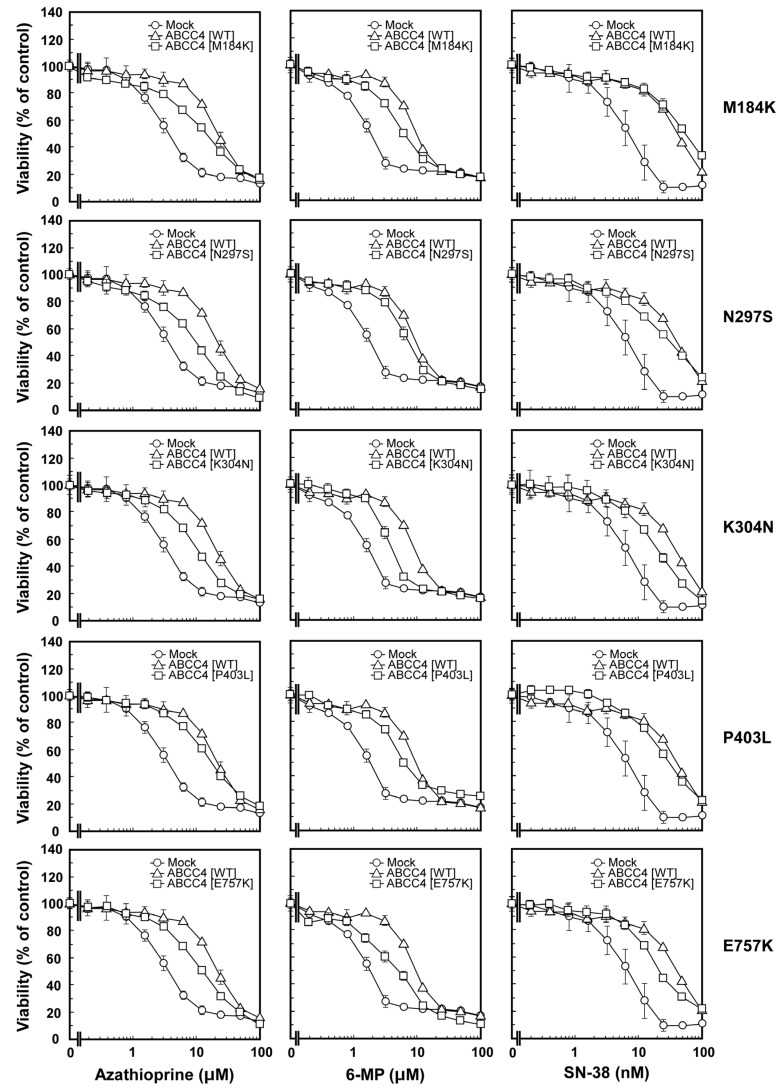
Anticancer drug resistance properties of the cells established using the Flp-In™ system. Anticancer drug resistance properties of cells were evaluated by using the 3-(4,5-Dimethyl-2-thiazol-2-yl)-2,5-diphenyl-2H-tetrazolium bromide (MTT) assay, as described in Materials and Methods. Data are expressed as mean values ± S.D. (*n* = 3–6).

We performed the 3-(4,5-Dimethyl-2-thiazol-2-yl)-2,5-diphenyl-2H-tetrazolium bromide (MTT) assay to determine and compare the anticancer drug resistance properties of the cells. As described in Materials and Methods, the drug resistance properties of the cells established using the Flp-In™ system were evaluated for seven anticancer drugs (Figure 4); the results are summarized in Table 2 as EC_50_ values. As shown in Table 2 and Figure 5, the cells expressing ABCC4 (WT) were more resistant to azathioprine, 6-mercaptopurine and SN-38 compared to the Flp-In-293/Mock cells. The EC_50_ values for azathioprine, 6-mercaptopurine and SN-38 showed that the cells expressing ABCC4 (WT) were 5.1-, 4.8- or 5.2-fold more resistant than the Flp-In-293/Mock cells, respectively (Table 2). In contrast, the resistance of cells expressing ABCC4 (WT) to all-transretinoic acid, etoposide, 5-fluorouracil and vincristine was comparable to that of Flp-In-293/Mock cells (Table 2).

As shown in Table 2 and Figure 5, cells expressing the SNP variants of ABCC4 (M184K, N297S, K304N, P403L or E757K) showed drug resistance, the levels of which differed with the cell types. Cells expressing the SNP variants of ABCC4 (M184K or N297S) were more resistant to azathioprine, 6-mercaptopurine and SN-38 compared to the Flp-In-293/Mock cells, where different resistance levels were observed compared to the cells expressing ABCC4 (WT) (Table 2 and Figure 5). Cells expressing the SNP variants of ABCC4 (K304N or E757K) were more resistant to azathioprine compared to the Flp-In-293/Mock cells, where their resistance levels were not as high as those of cells expressing ABCC4 (WT). EC_50_ values showed that the cells expressing ABCC4 (WT) were 1.2–1.7-fold more resistant to azathioprine than the cells expressing ABCC4 (M184K, N297S, K304N or E757K) (Table 2). The cells expressing ABCC4 (WT) were 1.4–2.0-fold and 1.9-fold more resistant to 6-mercaptopurine and SN-38 than those expressing the SNP variants of ABCC4 (K304N, P403L or E757K), respectively (Table 2 and Figure 5). In contrast, the drug resistance properties of cells expressing the SNP variants of ABCC4 (M184K, N297S, K304N, P403L or E757K) to all-trans retinoic acid, etoposide, 5-fluorouracil and vincristine were comparable to those of cells expressing ABCC4 (WT) and the Flp-In-293/Mock cells, except for the drug resistance property of cells expressing ABCC4 (K304N or P403L) to etoposide (Table 2).

## 3. Discussion

### 3.1. Establishment of Human *ABCC4*-Expressing Cells Using the Flp-In™ System

The drug resistance of cancer cells and the individual differences in their levels are usually caused by the overexpression of ABC transporters and SNPs in their genes, respectively [31]. Since the drug sensitivities of cells expressing the WT or SNP variants of ABCC4 have not been quantitatively evaluated so far, we performed a quantitative evaluation of the drug resistance profiles of cells expressing the WT or SNP variants of human ABCC4. Towards this objective, we established cells expressing the WT or non-synonymous SNP variants (M184K, N297S, K304N, P403L or E757K) of human ABCC4 using the Flp-In™ system, the Flp recombinase-mediated transfection system, which can integrate a single copy of cDNA into the FRT site in the telomeric region of the short arm of one copy of chromosome 12 in Flp-In-293 cells [27]. Although the integration site and the number of *ABCC4* cDNA were not determined in the present study, the levels of *ABCC4* mRNA were comparable among the cells transfected with the *ABCC4* cDNA (Figure 2). These results suggest that the transfected *ABCC4* cDNA was integrated into the designated site in the chromosome and that the number of integrated *ABCC4* cDNA was the same among the established cells.

### 3.2. Anticancer Drug Resistance Properties of Cells Established Using the Flp-In™ System

ABCC4 transports a broad spectrum of xenobiotics, including antiviral, antibiotic, antihypertensive and anticancer drugs such as azathioprine, 6-mercaptopurine and SN-38 [12,13,14,15,16,17,18,19,20,21,22,23,24,25]. Consistent with previous findings [19,20,21,22,24,25], cells expressing ABCC4 (WT) were more resistant to substrate drugs for ABCC4 (azathioprine, 6-mercaptopurine and SN-38) compared to the Flp-In-293/Mock cells, supporting that ABCC4 mediated resistance of the cells to these anticancer drugs. In contrast, the drug resistance profiles of cells expressing ABCC4 (WT) to non-substrate drugs for ABCC4 (all-trans retinoic acid, etoposide, 5-fluorouracil and vincristine) were comparable to those of the Flp-In-293/Mock cells, suggesting that ABCC4 does not mediate resistance of the cells to these anticancer drugs. These results indicated that the *ABCC4* cDNA transfected into the Flp-In-293 cells was functional and the effect of *ABCC4* SNPs on ABCC4-mediated drug resistance can be examined, although ABCC4 is intrinsically expressed in Flp-In™-293 cells (as shown in the present and previous studies [32]) and the transfected *ABCC4* cDNA may alter the expression of other ABC transporters.

Other researchers have previously shown that the non-synonymous SNPs in *ABCC4* altered the affinity of ABCC4 for its substrates in protein-based assays [13,24,25,26]. Banerjee et al. reported that non-synonymous SNPs in *ABCC4* altered the intracellular localization of ABCC4 in a cell-based assay [26]. However, the drug resistance properties of cells expressing the SNP variants of ABCC4 have never been examined in previous studies since it is difficult to establish cell lines suitable for this purpose using the traditional transfection system. In fact, the cells established by Banerjee et al. were prepared using the traditional transfection system with a pcDNA3.1 vector [26], where the integration number and integration site of the cDNA in the chromosome were not controlled. In contrast, in the present study, we established cells expressing the wild-type (WT) or SNP variants of human ABCC4 where the genomic integration number and integration site of the cDNA were regulated, and we showed for the first time that the drug resistance properties of cells expressing the SNP variants of ABCC4 were significantly different from those of cells expressing ABCC4 (WT).

The drug resistance properties of these cells are affected by the levels of ABC transporters as reported by Kosztyu et al. [33,34]. Previously, we showed that non-synonymous SNPs in *ABCG2* can affect the drug resistance properties of the cells by altering their substrate specificity, intracellular localization, or intracellular stability under conditions where the mRNA levels are comparable [27,29,35]. Banerjee et al. recently reported that non-synonymous SNPs in *ABCC4* (rs2274407, K304N or rs3765534, E757K) have altered ability of transporting substrates, as well as changed substrate specificity and membrane localization [26], which was also observed in the present study. Therefore, the findings of this study suggest that the non-synonymous SNP variants tested in the present study (M184K, N297S, K304N, P403L or E757K) increased intracellular accumulation of test drugs by altering substrate specificity, intracellular localization or intracellular stability of ABCC4 and resulted in altered drug sensitivity. Since measurement of the intracellular accumulation level of test drugs is important for understanding the molecular mechanism via which the non-synonymous SNP variants of ABCC4 alter the drug resistance profiles of cancer cells, this investigation is now in progress.

Taken together, in the present study, we first quantitatively evaluated the impact of non-synonymous *ABCC4* SNPs on the drug resistance profiles of cells expressing ABCC4 (WT or SNPs) and proposed an easy-to-use and quantitative approach for understanding the effect of SNPs on the drug resistance profiles of cancer cells. We suggest that the drug resistance profile can be altered by certain non-synonymous SNPs. On the contrary, clinical relevance of the present results remains to be investigated in cancer chemotherapy, and we primarily propose that retrospective studies be performed towards this objective. We strongly believe that the present study can improve our understanding of the effect of non-synonymous *ABCC4* SNPs on cancer chemotherapy and contribute to the development of novel therapeutic strategies for cancer treatment.

## 4. Materials and Methods 

### 4.1. Chemicals and Biological Reagents

The following reagents and drugs were purchased from the commercial sources indicated in parentheses: antibiotic-antimycotic cocktail solution, l-glutamine, high-glucose Dulbecco’s modified Eagle medium (DMEM), hygromycin B (Nacalai Tesque, Inc., Kyoto, Japan); fetal bovine serum (FBS) (Equitech-Bio, Inc., Kerrville, TX, USA); and 3-[4,5-dimethylthiazol-2-yl]-2,5-diphenyltetrazolium bromide (MTT reagent) (Sigma-Aldrich Co., St. Louis, MO, USA). SN-38 was generously provided by Yakult Honsha Co., Ltd. (Tokyo, Japan). All other chemicals used were of analytical grade.

### 4.2. Subcloning of Human *ABCC4* cDNA

The *ABCC4* (WT) cDNA in the pcDNA3.1(−) vector, kindly provided by Dr. Toshihisa Ishikawa (Tokyo Institute of Technology, Tokyo, Japan), was removed from the vector by NheI digestion. The resulting *ABCC4* cDNA was treated with alkaline phosphatase and ligated to the NheI sites of the pcDNA5/FRT expression vectors (Invitrogen, Life Technologies Corp., Carlsbad, CA, USA) using the Rapid DNA Dephos & Ligation Kit (Roche Diagnostics GmbH, Manheim, Germany) to prepare pcDNA5/FRT/*ABCC4* (WT) vectors.

### 4.3. Preparation of Plasmids Carrying the *ABCC4* SNP Variant cDNA

The pcDNA5/FRT expression vectors carrying the nonsynonymous SNP variants of *ABCC4* cDNA were prepared from the pcDNA5/FRT/*ABCC4* (WT) vectors. Data about the genetic polymorphic variants of *ABCC4* were obtained from the NCBI dbSNP database. In brief, nonsynonymous SNP variants of *ABCC4* were created by site-directed mutagenesis using the PrimeSTAR^®^ Max DNA Polymerase (Takara Bio Inc., Otsu, Japan); the primers are listed in Table 3. After polymerase chain reaction (PCR) (for 10 s at 98 °C, 12 cycles of reactions for 10 s at 98 °C, for 15 s at 55 °C and for 10 min at 72 °C), the reaction mixture was treated with Dpn I endonuclease for 1 h at 37 °C to degrade the original template plasmid pcDNA5/FRT/*ABCC4* (WT) vectors. The sequence of *ABCC4* cDNA in the resulting amplicons was confirmed by sequencing with Applied Biosystems 3130 and 3130 xl genetic analyzers (Applied Biosystems, Foster City, CA, USA).

### 4.4. Cell Culture

Flp-In™-293 cells (Invitrogen) were maintained in high-glucose DMEM supplemented with 10% (*v/v*) heat-inactivated FBS (Equitech-Bio, Inc., Kerrville, TX, USA), 4 mM l-glutamine, 100 U/mL penicillin, 100 μg/mL streptomycin, 250 ng/mL amphotericin B and 100 mg/mL zeocin in a humidified atmosphere of 5% (*v/v*) CO_2_ in air. The number of viable cells was determined using a hemocytometer and the trypan blue dye exclusion method. The Flp-In-293 cells were transfected with the pcDNA5/FRT or the pcDNA5/FRT/*ABCC4* expression vectors, and the Flp recombinase expression plasmid pOG44 using LipofectAmine™-2000 (Invitrogen) according to the manufacturer’s instructions. Colonies resistant to 50 mg/mL hygromycin B solution (Nacalai Tesque Inc., Tokyo, Japan) were picked and sub-cultured. The resulting cells that incorporated pcDNA5/FRT or pcDNA5/FRT/*ABCC4* were named as Flp-In-293/Mock or Flp-In-293/ABCC4 cells, respectively, and maintained in high-glucose DMEM supplemented with 10% (*v/v*) heat-inactivated FBS, 4 mM l-glutamine, 100 U/mL penicillin, 100 μg/mL streptomycin, 250 ng/mL amphotericin B and 50 mg/mL hygromycin B in a humidified atmosphere of 5% (*v/v*) CO_2_ in air.

### 4.5. Preparation of Total RNA and Synthesis of First-Strand cDNA

Cells were seeded into a 35-mm dish (TrueLine; density = 1 × 10^6^ cells/well) and pre-cultured for 3 days. Then, the cells were collected by pipetting with the culture medium into a 1.5-mL tube. This tube was centrifuged for 5 min at 4 °C at 300× *g*. The resulting cell pellets were rinsed twice with 1 mL of PBS (−) and suspended in 600 μL of the lysis buffer prepared from lysis/binding buffer (Roche Diagnostics GmbH) and stored at −80 °C until use for preparation of total RNA.

Total RNA was extracted from the cell lysates using the High Pure RNA isolation kit (Roche Diagnostics GmbH) according to the manufacturer’s instructions. The concentration of each extract was measured using the spectrophotometer DU640 (Beckman Coulter, Inc., Brea, CA, USA). Thereafter, the total RNA was reverse transcribed using the High-Capacity cDNA reverse transcription kits (Thermo Fisher Scientific Inc., Waltham, MA, USA) according to the manufacturer’s instructions. Concretely, 3–4.5 μg of the total RNA were used for reverse transcription reaction. As a reaction solution (50 μL), 10 × RT buffer (5 μL), 100 mM dNTP Mix (2 μL), 10 × RT Random Primers (5 μL), 50 U/μL MultiScribe™ Reverse Transcriptase (Thermo Fisher Scientific Inc.) (2.5 μL), 40 U/μL Recombinant RNase Inhibitor (1.25 μL), RT-PCR Grade Water and total RNA were mixed into a PCR tube, wherein the total volume was adjusted to 50 μL with RT-PCR Grade Water. The reverse transcription reaction consisted for 10 min at 25 °C, for 120 min at 37 °C and for 2 min at 95 °C.

### 4.6. Quantitative Real-Time PCR

*ABCC4* mRNA levels in the cells were quantitatively measured using GoTaq^®^ qPCR Master Mix, 2× (Promega KK., Tokyo, Japan) and gene-specific primers for *ABCC4* [forward primer (Takara Bio Inc.; 5095-085), reverse primer (Takara Bio Inc.; 5095-086)] and *GAPDH* (housekeeping gene), which was used as the internal control [forward primer (Takara Bio Inc.; 10000459), reverse primer (Takara Bio Inc.; 20000459)] by using Applied Biosystems 7500Fast Real-Time PCR System (Thermo Fisher Scientific Inc.). Concretely, the RNA quality used for qPCR was 1.50–1.70. As a reaction solution (20 μL), GoTaq^®^ qPCR Master Mix, 2 × (10 μL), 50 μM forward primer (0.08 μL), 50 μM reverse primer (0.08 μL), nuclease-free water (5.84 μL) and 2.5 ng/μL template cDNA (4 μL) were mixed each well in a 96-well plate. The Quantitative Real-Time PCR reaction consisted for 2 min at 95 °C, 40 cycles of reactions for 15 s at 95 °C, for 1 min at 60 °C, as a dissociation stage, for 15 s at 95 °C, for 1 min at 60 °C and for 15 s at 95 °C.

### 4.7. MTT Assay

Cells were seeded into 96-well plates (Thermo Fisher Scientific Inc.) at a density of 5 × 10^5^ cells/well and pre-cultured for 24 h. Then, the cells were exposed to different concentrations of the anticancer drugs for 72 h according to the results from previous studies [27,28,29,32], where the highest final concentrations of the anticancer drugs were 100 nM (SN-38 and vincristine), 1 μM (etoposide) or 100 μM (all-transretinoic acid, azathiopurine, 5-fluorouracil and 6-mercaptopurine), and the lowest was 0 M (control). After 72 h of treatment with each drug, the cells were incubated in the presence of 500 μg/mL MTT for 3 h. Thereafter, 100 μL of 20% sodium dodecyl sulfate (SDS) was added to each well, and the plates were incubated overnight at 37 °C in a humidified atmosphere of 5% (*v/v*) CO_2_ in air. The absorbance of formazan, a metabolite of MTT, in each well of the resulting solution was photometrically measured at 570 nm and at a reference wavelength of 630 nm using the Thermo Labsystems Multiskan Jax (Thermo Fisher Scientific Inc.). Cell viability was expressed as a percentage of the viability observed in the control group. Cytotoxicity was assessed with reference to the EC_50_ value, which was defined as the concentration needed for 50% reduction of survival based on the viability curve.

### 4.8. Preparation of Cell Lysates for Sodium Dodecyl Sulfate Poly-Acrylamide Gel Electrophoresis (SDS-PAGE)

Cells were seeded into a 35-mm dish (TrueLine) at a density of 1 × 10^6^ cells/well and pre-cultured for 3 days. After pre-culture, the cells were collected by pipetting with the culture medium into a 1.5-mL tube. The cells in the 1.5-mL tube were centrifuged for 5 min at 4 °C at 300× *g*. The resulting cell pellets were rinsed twice with 1 mL of PBS (−) and suspended in lysis buffer (50 mM Tris-HCl, pH 7.6, 5 mM EDTA, pH 8.0, 120 mM NaCl, 1% Triton X-100, 1 mM DTT, protease inhibitor and phosphatase inhibitor). Thereafter, the cell lysates were centrifuged for 10 min at 4 °C and 800× *g*. The resulting supernatant was retained, and the protein content of the samples was determined using the Bradford assay. After this assay, 50 μg of the supernatant protein were treated with PNGase F for 10 min at 37 °C.

### 4.9. SDS-PAGE and Western Blotting

Cell lysates samples were prepared independently from each cell in tripricate and 5 μg of them were mixed. The mixtures were fractionated using SDS-polyacrylamide gel electrophoresis (7.5%), and the gels were transferred to nitrocellulose membranes (GE Healthcare UK Ltd., Bucks, UK). These membranes were soaked in blocking buffer containing TBST (50 mM Tris-HCl, 150 mM NaCl, and 0.05% (*v/v*) Tween 20) containing 5% (*w/v*) skim milk powder at room temperature for >1 h, and incubated stationary overnight at 4 °C. The membranes were incubated with the monoclonal anti-ABCC4 antibody (M4I-10; GeneTex Inc., Alton Parkway Irvine, CA, USA); 1:1000 dilution in TBST containing 5% (*w/v*) skim milk powder) or anti-GAPDH antibody (anti-GAPDH-Clone 6C5 mouse monoclonal, igG2b; American Research Products, Inc., Waltham, MA, USA; 1:1000 dilution in TBST containing 5% (*w/v*) skim milk powder) for 1 h at room temperature with shaking after rinsing with TBST. Thereafter, the membranes were washed with TBST, followed by incubation with the secondary antibody for 1 h at room temperature with shaking. The secondary antibody for ABCC4 is rabbit anti-Rat IgG:HRP (Enzo Life Sciences, Inc., Farmingdale, NY, USA; 1:1000 dilution in TBST containing 5% (*w/v*) skim milk powder), and the secondary antibody for GAPDH is anti-mouse IgG, HRP-linked antibody (Cell Signaling Technology, Inc., Danvers, MA, USA; 1:1000 dilution in TBST containing 5% (*w/v*) skim milk powder). The blots were developed using Western Lighting Chemiluminescent Reagent Plus (PerkinElmer Life and Analytical Sciences, Boston, MA, USA) and detected using WSE-6100 LuminoGraph I (ATTO CORPORATION, Tokyo, Japan). The signal intensities derived from ABCC4 or GAPDH were measured using ImageJ (Wayne Rasband, Bethesda, MD, USA).

### 4.10. Statistical Analysis

Statistical analyses were performed using JSTAT version 20.0J (Masato Sato, Japan), which is a software for statistical Tukey HSD analysis. A *p* value of <0.01 (MTT assay) or <0.05 (western blotting) was considered statistically significant.

## Figures and Tables

**Table 1:** Summary of the non-synonymous SNPs in *ABCC4* selected in the present study.

Variant	rsNumber	Nucleotide Position	Nucleotide Change	Amino Acid Position	Amino Acid Change
M184K	rs45454092	551	t > a	184	Met > Lys
N297S	rs200387797	890	a > g	297	Asn > Ser
K304N	rs2274407	912	g > t	304	Lys > Asn
P403L	rs11568705	1208	c > t	403	Pro > Leu
E757K	rs3765534	2269	g > a	757	Glu > Lys

Data on genetic polymorphic variants of *ABCC4* were obtained from the the National Center for Biotechnology Information (NCBI) dbSNP database.

**Table 2:** Anticancer drug resistance profiles (EC_50_) of the cells.

Compounds	EC_50_						
	Mock	WT	M184K	N297S	K304N	P403L	E757K
ATRA (μM)	40.0 ± 10.1	42.1 ± 4.8	43.4 ± 3.2	48.1 ± 8.0	40.3 ± 4.8	41.0 ± 2.9	42.0 ± 4.3
Azathioprine (μM)	4.2 ± 1.0	21.5 ± 1.0 *	14.3 ± 1.8 *^,^**	12.6 ± 2.4 *^,^**	12.6 ± 2.2 *^,^**	17.9 ± 1.0 *	15.2 ± 3.3 *^,^**
Etoposide (nM)	204.9 ± 16.2	256.6 ± 31.7	210.3 ± 13.2	254.1 ± 28.2	326.3 ± 33.6 *	321.0 ± 14.6 *	208.4 ± 17.9
5-FU (μM)	7.7 ± 1.4	6.3 ± 2.2	11.1 ± 1.4	4.5 ± 1.2	11.0 ± 2.7	4.3 ± 1.0	5.9 ± 1.2
6-Mercaptopurine (μM)	1.9 ± 0.4	9.1 ± 1.8 *	6.4 ± 0.7 *	6.6 ± 0.9 *	4.6 ± 0.3 **	6.4 ± 0.5 *	5.1 ± 1.4 **
SN-38 (nM)	8.7 ± 1.1	45.5 ± 10.2 *	50.2 ± 6.6 *	29.5 ± 8.5 *	22.5 ± 0.3 **	24.4 ± 7.4 **	21.6 ± 1.6 **
Vincristine (nM)	2.9 ± 1.1	1.6 ± 0.5	2.1 ± 0.7	2.1 ± 0.8	2.3 ± 0.4	1.9 ± 0.4	1.7 ± 0.3

Definitions: ATRA, All-trans-retinoic acid; 5-FU, 5-Fluorouracil; SN-38, 7-Ethyl-10-hydroxy-camptothecin. The drug resistance properties of cells established using the Flp-In™ system were evaluated, as described in Materials and Methods. Data are expressed as mean values ± S.D. (*n* = 3–6). Statistical analyses for significance were performed using One-way ANOVA and Tukey HSD test (* *p* < 0.01 compared to the Mock group; ** *p* < 0.01 compared with the wild type (WT)).

**Table 3:** Polymerase chain reaction (PCR) primers and conditions for site-directed mutagenesis to generate genetic polymorphic variants of *ABCC4*.

Variant	Forward/Reverse (F/R) Primers	Primer Sequence (5′→3′)	Primer Length (Bases)	% GC	*T*_m_ (°C)
M184K	F	CACTTCGTCTTAGTAACA**A**GGCCATGGGGAAGACAAC	37	48.6	80.5
	R	GTTGTCTTCCCCATGGCC**T**TGTTACTAAGACGAAGTG			
N297S	F	GCCTGGGAAAAGTCATTTTCAA**G**TCTTATTACCAATTTGAGAAAG	45	35.6	78.9
	R	CTTTCTCAAATTGGTAATAAGA**C**TTGAAAATGACTTTTCCCAGGC			
K304N	F	TCTTATTACCAATTTGAGAAA**T**AAGGAGATTTCCAAGATTCTGAG	45	31.1	77.9
	R	CTCAGAATCTTGGAAATCTCCTT**A**TTTCTCAAATTGGTAATAAGA			
P403L	F	CGCAACCGTCAGCTGC**T**GTCAGATGGTAAAAAGATG	36	50.0	80.5
	R	CATCTTTTTACCATCTGAC**A**GCAGCTGACGGTTGCG			
E757K	F	AAATGGAGGAGGAAATGTAACC**A**AGAAGCTAGATCTTAACTGGTA	45	37.8	79.7
	R	TACCAGTTAAGATCTAGCTTCT**T**GGTTACATTTCCTCCTCCATTT			

Sites of mutagenesis are underlined. % GC indicates the guanine and cytosine content in the PCR primer set. *T*_m_ is the melting temperature for each PCR primer set.

## Abbreviations

ABC	ATP-binding cassette
ABCC4	ATP-binding cassette subfamily C member 4
BSA	bovine serum albumin
DMEM	high-glucose Dulbecco’s modified Eagle’s medium
DTT	dithiothreitol
EC_50_	half maximal (50%) effective concentration
FBS	fetal bovine serum
FRT	Flp recombination target
GAPDH	glyceraldehyde-3-phosphate dehydrogenase
HRP	horseradish peroxidase
MTT	3-(4,5-dimethyl-2-thiazol-2-yl)-2,5-diphenyl-2H-tetrazolium bromide
MRP	multi drug resistance protein
NCBI	National Center for Biotechnology Information
PBS(−)	phosphate-buffered saline without both Ca^2+^ and Mg^2+^
PCR	polymerase chain reaction
qPCR	quantitative real-time PCR
SDS-PAGE	sodium dodecyl sulfate poly-acrylamide gel electrophoresis
SN-38	7-Ethyl-10-hydroxy-camptothecin
SNP	single-nucleotide polymorphism
TBS	Tris-buffered saline
TBST	TBS with 0.05% (*v/v*) Tween 20
WT	wild type
